# Ultrablack color in velvet ant cuticle

**DOI:** 10.3762/bjnano.15.122

**Published:** 2024-12-02

**Authors:** Vinicius Marques Lopez, Wencke Krings, Juliana Reis Machado, Stanislav Gorb, Rhainer Guillermo-Ferreira

**Affiliations:** 1 Lestes Lab, Federal University of Triângulo Mineiro, Uberaba, Minas Gerais, Brazil; 2 Department of Functional Morphology and Biomechanics, Kiel University, Am Botanischen Garten 1–9, 24098 Kiel, Germany; 3 Department of Cariology, Endodontology and Periodontology, Universität Leipzig, Liebigstraße 12, 04103 Leipzig, Germany; 4 Institute of Biological and Natural Sciences, Federal University of Triângulo Mineiro, Uberaba, Minas Gerais, Brazil

**Keywords:** animal coloration, biophotonics, Hymenoptera, insects, Mutillidae, superblack, surface

## Abstract

We studied the ultrastructure of the ultrablack cuticle in *Traumatomutilla bifurca*, an enigmatic and visually striking species of velvet ants (Hymenoptera, Mutillidae). Using a combination of scanning electron microscopy (SEM), transmission electron microscopy (TEM), confocal laser scanning microscopy (CLSM), and optical spectroscopy, we conducted a comprehensive analysis of the cuticle to elucidate its unique optical properties. SEM imaging provided a detailed surface morphology, while TEM provided insights into the internal structure. CLSM showed that the cuticle exhibits no autofluorescence. Our findings reveal a highly specialized cuticle, characterized by microstructures that effectively minimize reflectance and enhance light absorption. Optical spectrometry confirmed the ultrablack nature of the cuticle, with the measured reflectance approaching minimal levels across a broad spectrum of wavelengths. Therefore, our study contributes to a deeper understanding of ultrablack biological materials and their potential applications in biomimetics.

## Introduction

The phenomenon of highly absorptive colors, also known as ultrablack, has risen considerable interest in recent years because of its potential applications in various fields, including optics, camouflage, and solar energy harvesting [1–2]. These colors are characterized by their ability to reflect an exceptionally low amount of visible light. Inspired by several biological examples observed in some organisms, scientists are committed to unraveling the mechanisms underlying the development of ultrablack technical surfaces, seeking to replicate such structures in synthetic and natural materials with equivalent properties [3–8].

**Figure 1:**
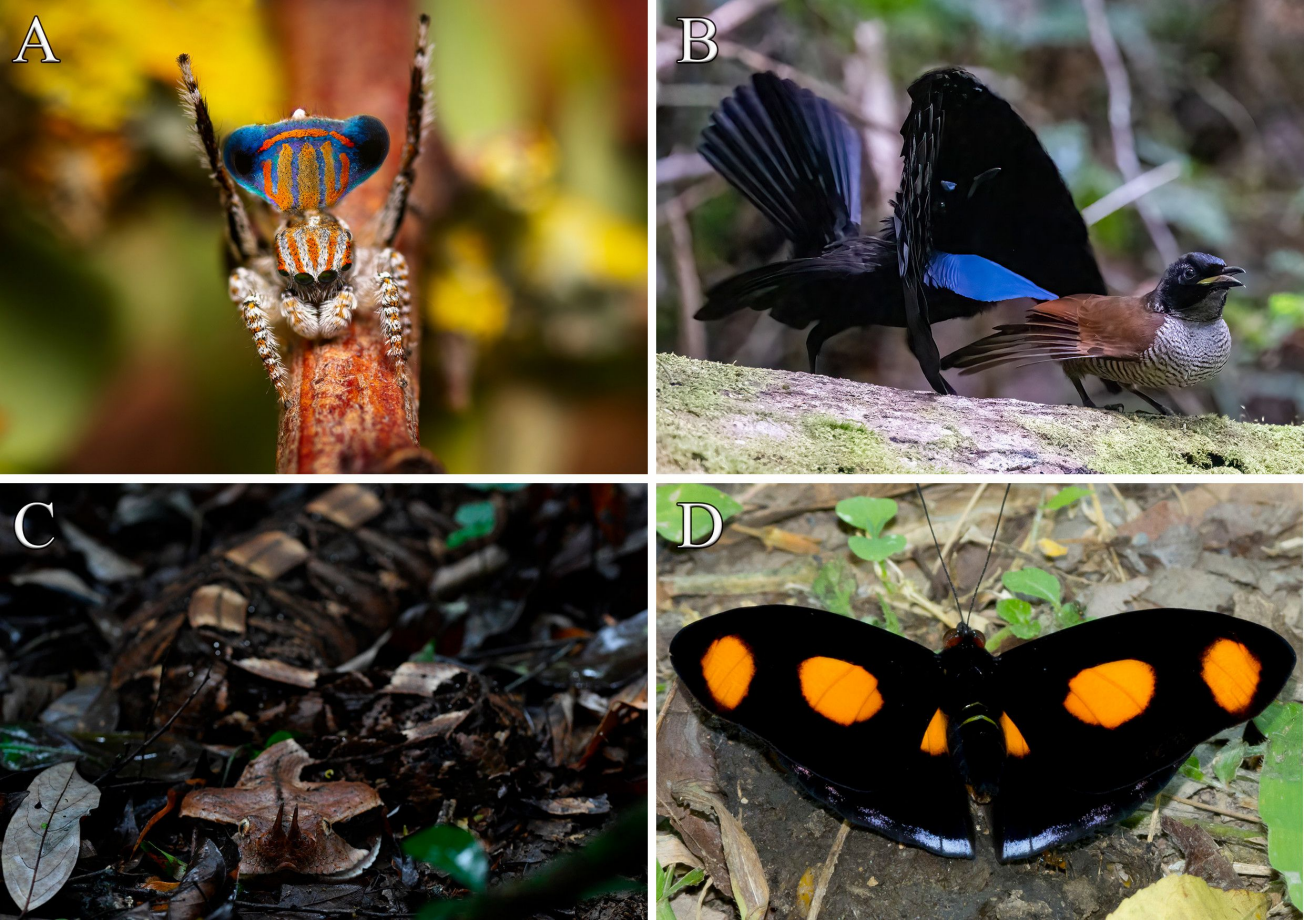
Examples of ultrablack colors in animals within their natural habitats. (A) Peacock spider (*Maratus tasmanicus*, Araneae: Salticidae) (© 2021 Henning Kallies), (B) bird-of-paradise (*Lophorina niedda*, Passeriformes: Paradisaeidae) (© 2022 Jes Lefcourt), (C) Gaboon viper (*Bitis rhinoceros*, Squamata: Viperidae) (By Justin Philbois), and (D) butterfly (*Catonephele numilia,* Lepidoptera: Papilionidae). Figures A and B were reproduced from https://www.inaturalist.org/observations/95228671 and https://www.inaturalist.org/observations/13984541, respectively, with permission from the respective authors. This content is not subject to CC BY 4.0. Figures C and D were reproduced from https://www.inaturalist.org/observations/131996241 and https://www.inaturalist.org/photos/17218853, respectively (published by iNaturalist, distributed under the terms of the Creative Commons CC0 1.0 Universal license).

Ultrablack colors are a rare spectacle among animals. These colors with high absorption are formed in nature by a sophisticated arrangement of microstructures (i.e., structures visible under microscope) alongside pigment depositions in underlying tissues [9–10]. For instance, in male peacock spiders (Figure 1A), ultrablack pigmentation originates from the interaction of light with arrays of microlenses on the cuticle or overlapping brush-like scales positioned just above a densely pigmented absorbing layer. Similar phenomena can be observed in butterflies [11–12], birds [13], and snakes [14–15]. This intricately structured setup prolongs the light’s exposure to the melanized integument, thereby augmenting light absorption by the pigment [10]. Consequently, ultrablack colors exhibit an extraordinarily low reflectance across ultraviolet and visible (UV–vis) spectrum wavelengths, often falling below 0.5% of the incident light [10,13].

The ultrablack surfaces found in certain organisms present a remarkable adaptation shaped by selective pressures in their respective environments. For example, combining conspicuous visual cues with ultrablack colors may provide heightened internal visual contrast, thus highlighting sexually selected colors in peacock spiders [10,13] (Figure 1A) and birds-of-paradise (Figure 1B). Likewise, ultrablack colors may offer thermoregulatory and disruptive advantages to vipers (Figure 1C) and assist butterflies in predator evasion (Figure 1D) [9,15]. Therefore, the parallel between ultrablack colors in animals underscores the convergent evolution of anti-reflective mechanisms as an important strategy for survival and reproductive success across diverse habitats and ecological contexts.

The evolution of ultrablack colors in animals highlights nature’s ingenuity in achieving structurally assisted absorption to reduce specular reflectance. This demonstrates how organisms have developed sophisticated mechanisms to modulate the interaction between light and biological surfaces, resulting in highly absorptive and minimally reflective colors. These adaptations play a role in animal survival and reproductive success, offering substantial adaptive advantages within their respective habitats.

Velvet ants (Hymenoptera: Mutillidae) are wasps that exhibit a wide range of colors, usually contrasting with black integument. Their coloration is considered to be aposematic, that is, colors that ward off predators, but also may have some function in camouflage and protection against solar radiation [16]. Although hymenopterans (sawflies, bees, wasps, and ants) are one of the most diverse animal groups, few studies have addressed the mechanisms behind their coloration. Here, we present the discovery of the ultrablack cuticle in the velvet ant *Traumatomutilla bifurca* (Klug, 1821), a feature previously unreported in the Hymenoptera.

## Materials and Methods

### Study species

**Figure 2:**
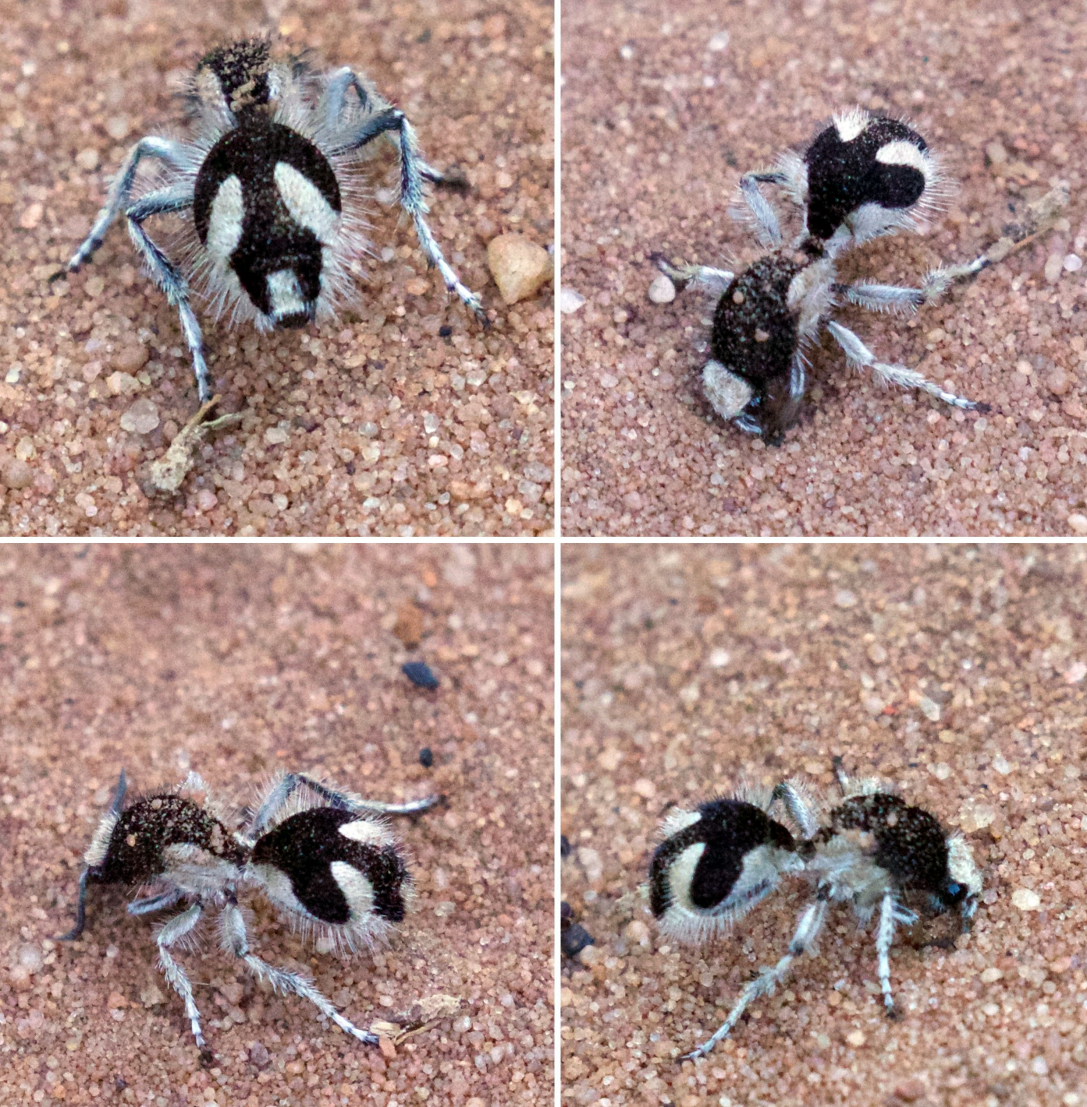
Velvet ants (*Traumatomutilla bifurca*, Hymenoptera: Mutillidae) in their natural habitats. The images highlight the dark integument with contrasting patterns of black and white setae. The photos depicted in Figure 2 were adapted (cropped) from https://www.inaturalist.org/photos/62624578, https://www.inaturalist.org/photos/62624589, https://www.inaturalist.org/photos/62624582, and https://www.inaturalist.org/photos/62624568 (© 2020 shrike2, published by iNaturalist, distributed under the terms of the Creative Commons Attribution-Non Commercial 4.0 International License, https://creativecommons.org/licenses/by-nc/4.0/). This content is not subject to CC BY 4.0.

*Traumatomutilla bifurca* (Hymenoptera: Mutillidae) is a species widely distributed in Brazil, primarily in Brazilian savanna and Caatinga areas [17]. Distinguished by its black integument adorned with contrasting patterns of black and white setae along its body (Figure 2), this species exhibits behavior akin to other females of the family. Frequently observed walking on exposed sandy soil, often in aggregations of bees (personal observation), *T. bifurca* also occurs in forested habitats adjoining open landscapes. Demonstrating remarkable mobility, it covers significant distances in pursuit of hosts on the ground. While sightings may occur throughout the day, peak activity typically coincides with the early morning and late afternoon, during periods of subdued sunlight (personal observations). For subsequent analyses, we utilized female specimens collected from Caatinga regions in Pernambuco, Brazil (09°19’44.2’’S, 42°33’30’’W) in February 2022. The specimens were preserved in absolute alcohol.

### Scanning electron microscopy (SEM)

To investigate the internal characteristics of cuticle cross sections, we prepared the samples by sectioning, allowing for the visualization of the underlying structures. Prior to SEM imaging, a thin layer of gold–palladium, 10 nm thick, was applied to the samples. We utilized a Hitachi S-4800 SEM, operating at 3 kV. Images were captured at different magnifications, starting at 15,000× and adjusted as needed.

### Transmission electron microscopy (TEM)

TEM was utilized to examine the internal cuticle morphology at high resolution at a nanometer scale. The apparatus was configured to operate at 50 kV with a minimum vacuum column pressure of 5.10–2.00 hPa. For sample preparation, transversely sectioned *T. bifurca* specimens were fixed in Karnovsky fixative solution + 0.1% ruthenium red for 12 h. Following buffer washing, a post-fixative solution (osmium tetroxide + 0.1% ruthenium red) was applied at room temperature. After rinsing with alcohol, dehydration proceeded through sequential baths of 70%, 90%, and three changes of 100% alcohol. The material (meso and metasoma) was embedded in epoxy resin, and the molds were polymerized at 60 °C. After block trimming, ultrathin sections (60 to 80 nm) were obtained using a Leica EM UC7 ultramicrotome (EM UC7, Leica Biosystems, Solms, Germany) and contrasted with osmium tetroxide.

### Confocal laser scanning microscopy (CLSM)

The capacity of insect cuticle to emit autofluorescence at various wavelengths is extensively documented, influenced by material composition, degree of sclerotization, and the presence of resilin [18]. Insect cuticle with protein-dominated regions, which can include resilin, an elastic protein present in arthropod cuticles, or unsclerotized chitin exhibits autofluorescence when excited with a laser of 405 nm wavelength. Regions with a low degree or high degree of sclerotization emit autofluorescence when excited with lasers of 488 nm (low degree) or 555 and 639 nm wavelengths (both high degree).

For documentation of the natural autofluorescence, cuticle was cut and arranged on glass slides. Each piece was enclosed by multiple reinforcement rings and then filled with glycerine (99.5% or higher purity, water-free, Carl Roth GmbH & Co. KG, Karlsruhe, Germany) and covered with a glass slip. Following the method described in [10], we documented the samples using a Zeiss LSM 700 confocal laser scanning microscope (Carl Zeiss Microscopy GmbH, Jena, Germany). Four stable solid-state lasers emitting at 405, 488, 555, and 639 nm wavelengths were utilized. Specific bandpass or longpass emission filters (ranging from 420–480 nm, 490 nm or higher, 560 nm or higher, and 640 nm or higher) were employed accordingly. After scanning, the autofluorescence images were combined (using maximum intensity projection) with Zeiss Efficient Navigation (ZEN) software (Carl Zeiss MicroImaging GmbH, Jena, Germany). We assigned blue to the autofluorescence signal from the 405 nm laser, green to the 488 nm laser, and red (50% saturation) to both the 555 and 639 nm lasers.

### Optical spectrometry

Reflectance spectra measurements were conducted using a high-resolution optical fiber spectrometer (Flame; Ocean Insight, Inc., Dunedin, FL, USA), equipped with a DH-2000-BAL light source (Ocean Insight, Inc.) and an optical fiber probe consisting of a 400 µm detector and a light guide. Spectral luminance was controlled using a Spectralon^®^ standard that reflects 99% of light in the UV–vis spectrum. A standard distance of 5 mm was maintained between the fiber probe and both the Spectralon standard and the sample. This distance was determined using the scale provided on the Ocean Insight holder, ensuring consistency across all measurements. Spectra were obtained with normal incidence, covering the spectral range from 300 to 800 nm, with light focused on two parts of the body, namely, the black thorax and the black spot on the metasoma. For each analyzed individual (*N* = 3), five replicates were measured for each analyzed body part, totaling to 60 reflectance spectra.

The average reflectance spectra of the ultrablack colors of the velvet ant female were then compared with spectra from animal species with the lowest reflectance in nature, that is, the butterfly *Troides helena* (Papilionidae), the peacock spider *Maratus karrie* (Araneae: Salticidae), and the bird-of-paradise *Drepanornis bruijnii* (Passeriformes: Paradisaeidae). The spectra of these species were obtained from data provided in [10,12], and species with the highest absorbance in the UV–vis spectrum were selected.

### Thermal images

This experimental protocol was adapted from [15]. For the thermal images, a velvet ant specimen was carefully positioned on a polystyrene plate, serving as a thermal insulator, and covered with a layer of sand measuring 2 cm in thickness. Using a Fluke TiS75+ Thermal Camera, thermal images were captured before, during, and after a controlled heating process induced by a thermal lamp positioned 10 cm away. Prior to initiating the experiment, the female specimen was photographed, and subsequent images were taken at 1 min and 2 min intervals during the heating process. Throughout the experiment, the ambient temperature was meticulously maintained at a controlled 24 °C.

## Results

**Figure 3:**
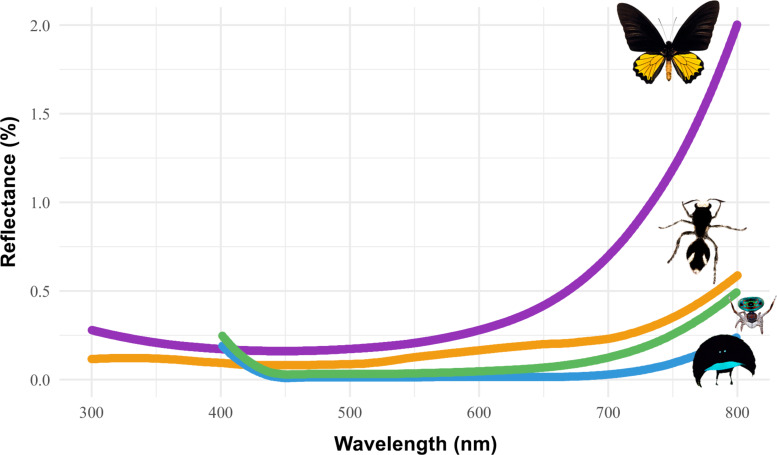
Reflectance spectra of ultrablack colors in the velvet ant *Traumatomutilla bifurca* (Hymenoptera: Mutillidae) (orange line) (original data), the butterfly *Troides helena* (Lepidoptera: Papilionidae) (purple line), the peacock spider *Maratus karrie* (Araneae: Salticidae) (green line), and the bird-of-paradise *Drepanornis bruijnii* (Passeriformes: Paradisaeidae) (blue line). The spectra of the spider and bird were obtained from [10] and that of the butterfly was obtained from [12]. The spectra of *T. bifurca* are derived from our study. The illustrations of velvet ant and bird-of-paradise were created using Adobe Photoshop. Butterfly image is from Insectpedia - stock.adobe.com. This content is not subject to CC BY 4.0. The image of the peacock spider was reproduced from https://www.inaturalist.org/observations/160699324 (© 2022 Donna Newton, published by iNaturalist, distributed under the terms of the Creative Commons Attribution-Non Commercial 4.0 International License, https://creativecommons.org/licenses/by-nc/4.0/). This content is not subject to CC BY 4.0.

Our results indicate that the cuticle reflectance of *T. bifurca* females closely resembles the spectra of other animal species with ultrablack coloration (Figure 3). The ultrablack colors in *T. bifurca* are less reflective compared to the butterfly *T. helena* and are similar to those seen in peacock spiders and birds-of-paradise (Figure 3).

The primary characteristic of the ultrablack cuticle is its pronounced black coloration, as evidenced by the photographs and CLSM images. This intense black color is likely attributed to the presence of melanin. Interestingly, unlike the melanin described in *Hermetia illucens* [19], the melanin in *T. bifurca* does not exhibit autofluorescence. This distinction and the fact that all other structural features serve to enhance this fundamental black coloration are noteworthy.

**Figure 4:**
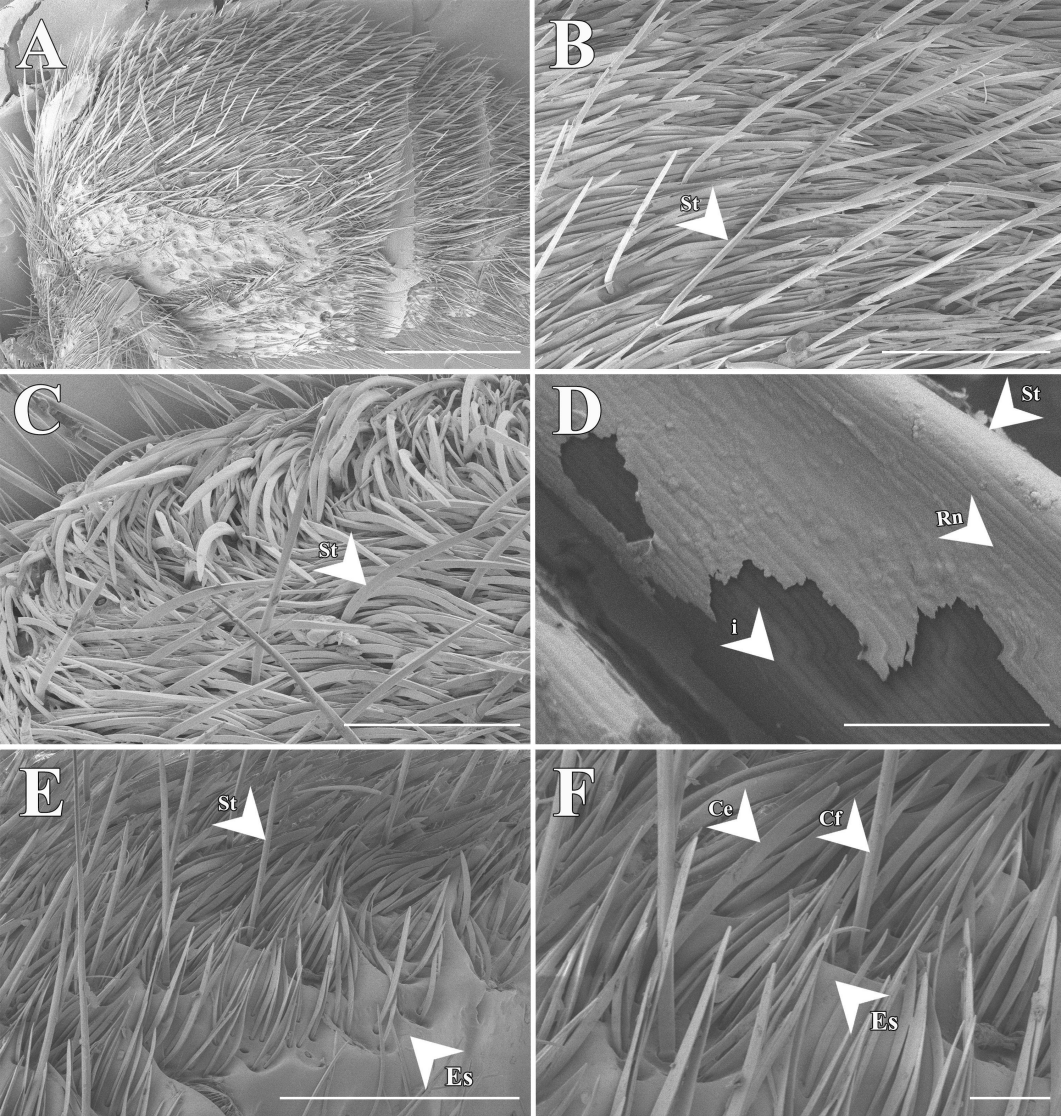
Scanning electron microscopy (SEM) images of the cuticle surface of *Traumatomutilla bifurca* (Hymenoptera: Mutillidae). It exhibits a dense covering of spines and setae (A–F). The setae display an arrangement of slightly flattened filamentous structures (B, C, E, and F). The setae feature grooved nanostructure and are hollow (D), with no morphological distinction between white (B and C) and black (E and F) setae. Legend: St = setae; Rn = grooves on the bristles; i = hollow interior of the setae; Es = surface sculpturing; Ce = slightly flattened setae; Cf = spines. Scale bars: 1 mm in A, 400 µm in B, 200 µm in C, 4 µm in D, and 500 µm in E, 100 µm in F.

SEM analysis of the cuticle surface in *T. bifurca* reveals a dense covering of spines and setae (Figure 4). The setae display nanostructures in the form of grooves and are hollow (Figure 4D). No morphological distinction was observed between white and black setae (Figure 4A).

**Figure 5:**
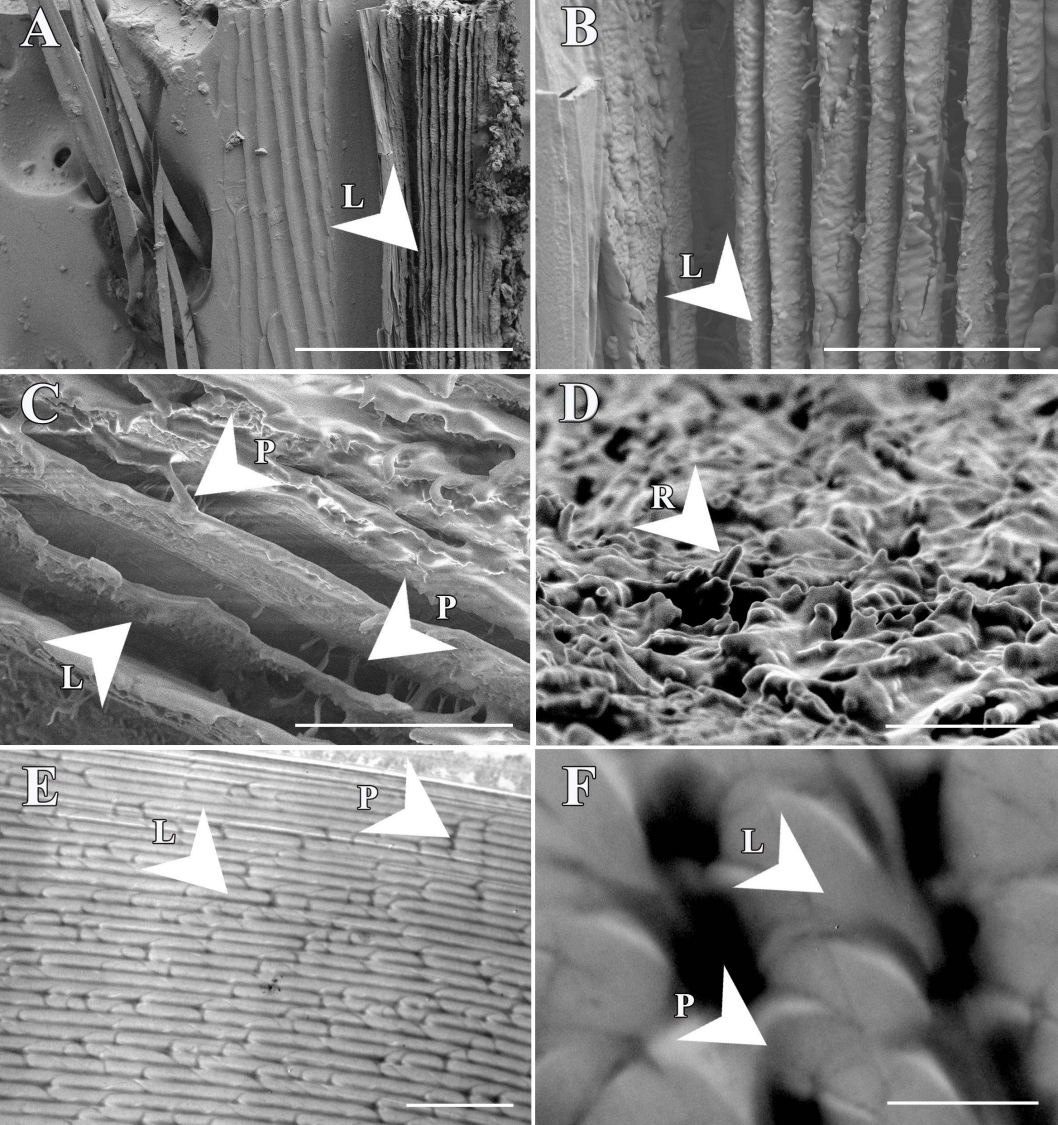
Scanning (A–D) and transmission (E and F) electron microscopy images of the cuticle structure of *Traumatomutilla bifurca* (Hymenoptera: Mutillidae). There is a complex structural arrangement of lamellar units (C and D), stacked on top of each other (E and F). There are also dark transverse bands that interconnect two consecutive longitudinal bands of the same lamellae (arrow), or of two separate lamellae (A and B). Legend: L = lamellae; P = pillars; R = rugosity of chitin fibers embedded in a protein matrix. Scale bars: 100 µm in A, 20 µm in B, 15 µm in C, 3 µm in D, 5 µm in E and 1 µm in F.

**Figure 6:**
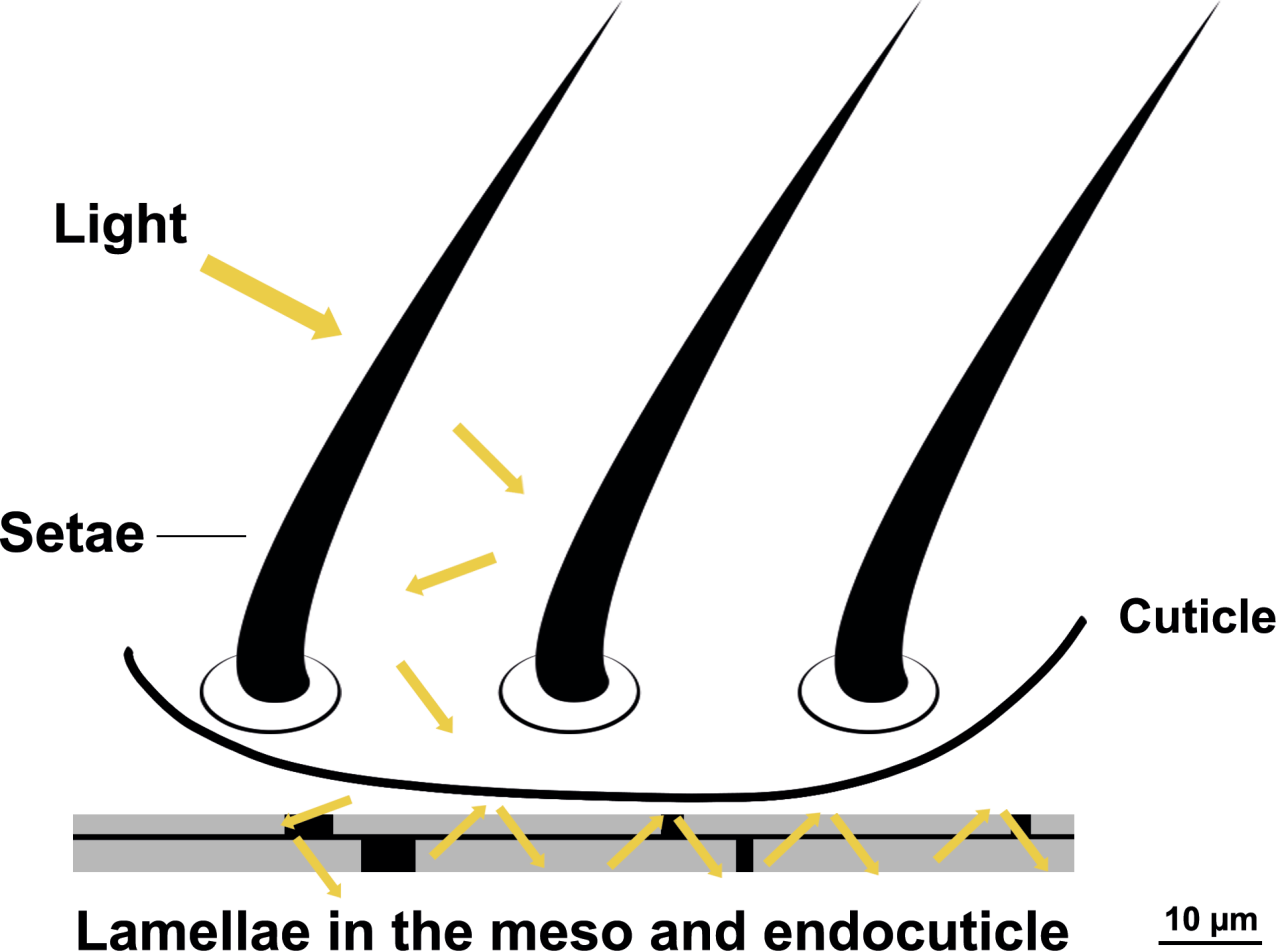
Scheme illustrating the functionality of the surface structure in *Traumatomutilla bifurca* (Hymenoptera: Mutillidae). Proposed mechanisms of structurally assisted absorption by surface sculpturing and setae, as light (yellow arrows) traverses through the dark pigmented cuticle and into the absorbing layer. Additionally, there is multiple scattering between protrusions and iterative absorption as light traverses through the cuticle at each lamella. Thus, the increased path length of light through lamellae may cause enhanced absorption.

**Figure 7:**
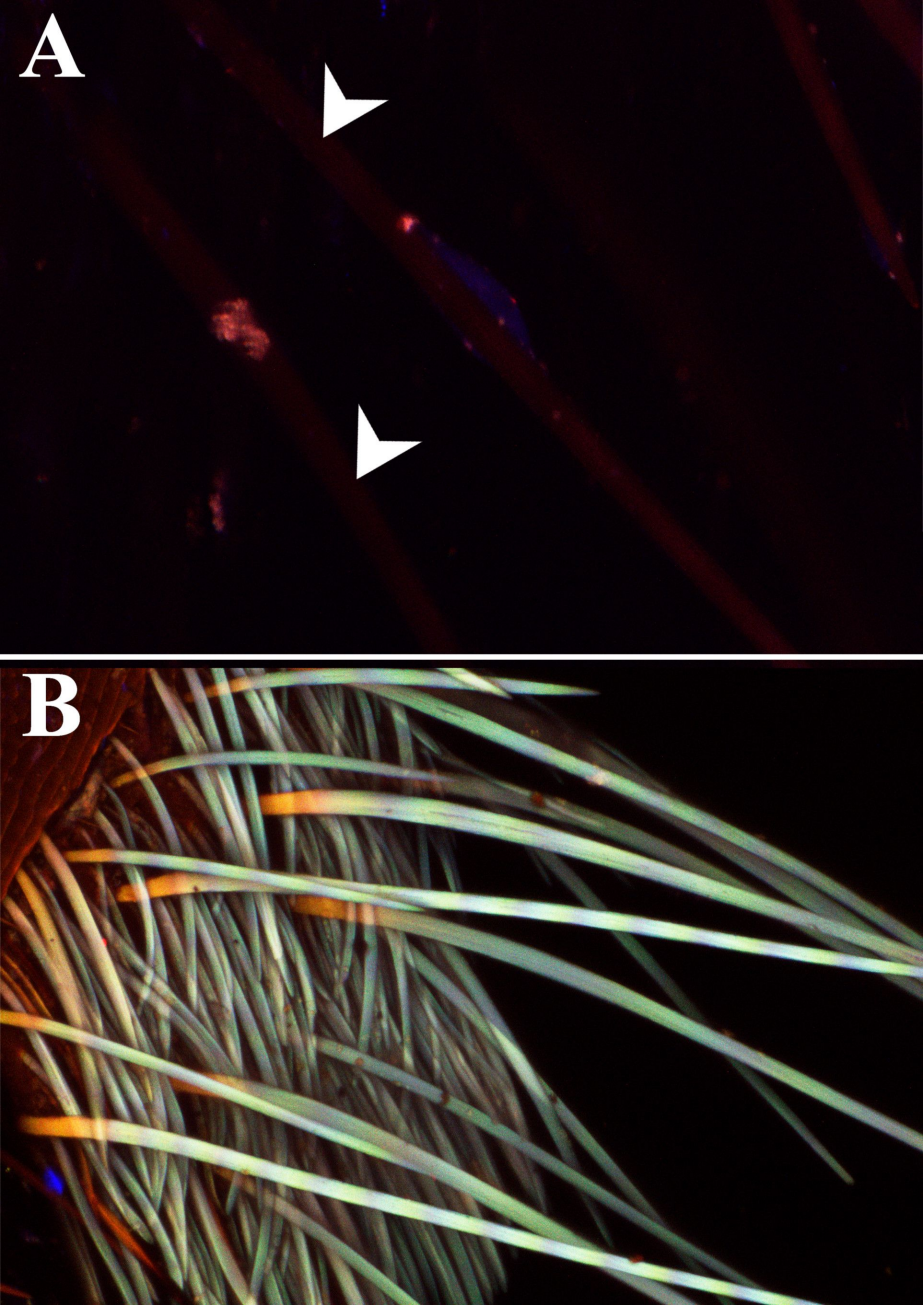
Confocal laser scanning microscopy micrographs (maximum intensity projections) showing different types of autofluorescence exhibited by the cuticle. Blue regions contain resilin or some other proteins, while green, orange, and red structures represent different degrees of sclerotization. Black regions are presumably melanized. (A) The cuticle of the velvet ant *Traumatomutilla bifurca* shows no autofluorescence, while black setae show a very low signal (white arrows). (B) The autofluorescence in the white setae is whitish, which means that all of the wavelengths are present in the signal.

The cuticle is composed of overlapping lamellae with connective pillars and underlying layers (Figure 5). The SEM and TEM images of the *T. bifurca* cuticle reveal that the cuticle sculpturing and setae together with the black pigment may facilitate structural light absorption (Figure 5). The setae in Figure 4, with their grooved nanostructures and hollow interiors, and the stacked lamellae (see L in Figure 5) are integral to the structures represented in Figure 6, where setae and lamellae likely enhance light absorption through multiple scattering and increased path length as light interacts with the cuticular protrusions and lamellar layers. Additionally, iterative scattering and absorption occur between the cuticular protrusions and the underlying layer with presumable absorptive properties (Figure 6). This process increases the path length through lamellae, leading to more efficient absorption, as evidenced by CLSM images of the target species (Figure 7).

**Figure 8:**
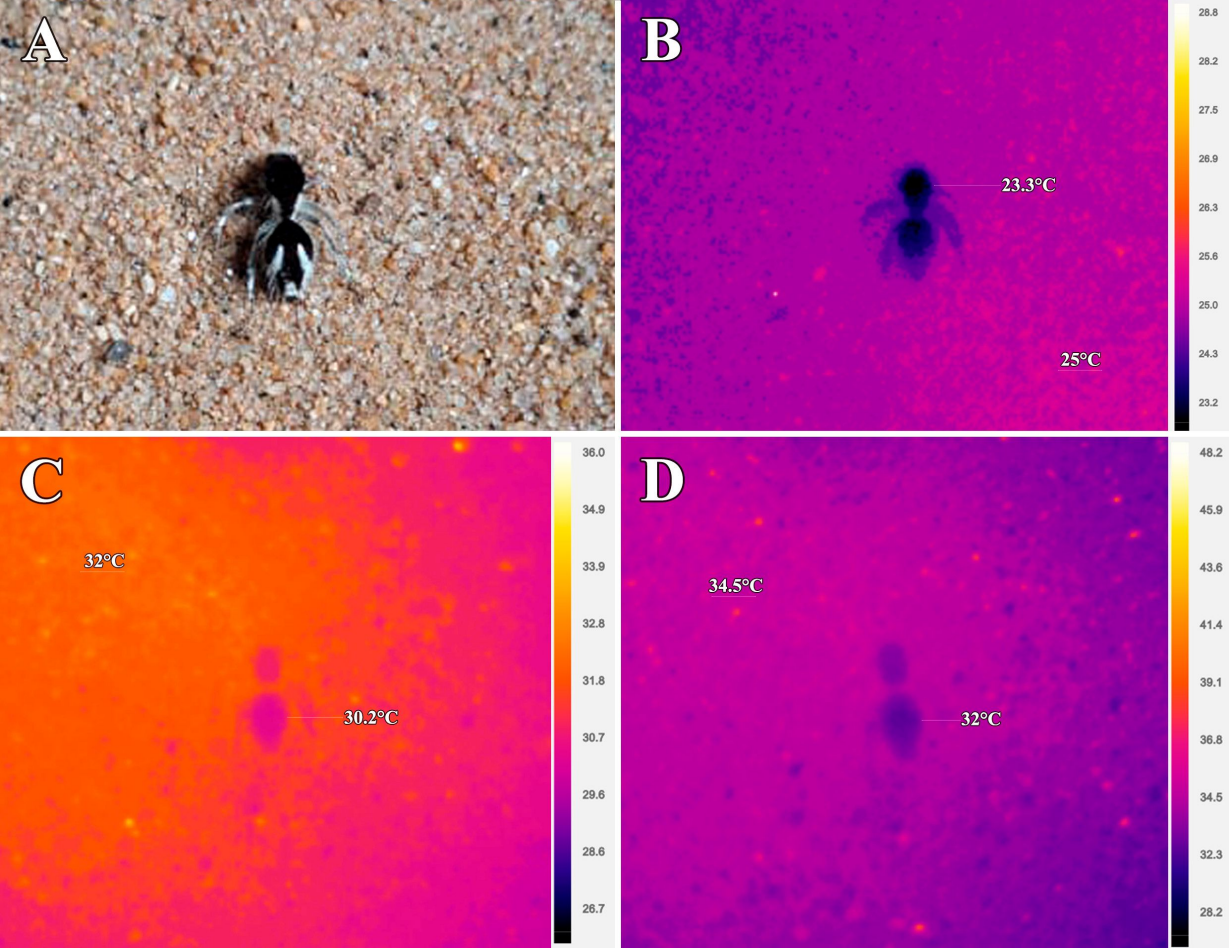
Thermal images of a female *Traumatomutilla bifurca* (Hymenoptera: Mutillidae). (A) Visible spectrum image, (B) thermal image of the animal before heating, (C) thermal image after 1 min of heating, and (D) thermal image after 2 min of heating.

Thermal imaging results indicate that *T. bifurca* was consistently 2 °C cooler than the ambient temperature (Figure 8). Furthermore, no thermal differences were observed between areas with white hair and ultrablack cuticle (Figure 8). This is the evidence that the color plays a secondary role in thermobiology of this animal; rather, the isolation properties of the hair cover are decisive for thermoregulation function.

## Discussion

The results suggest that the velvet ant *Traumatomutilla bifurca* possesses an ultrablack, structurally intricate antireflective coloration. The ultrablack coloration is produced by structurally assisted light guiding and absorption due to complex arrangements of microstructures at the cuticle surface above absorptive lamellae. The surface microstructure of the ultrablack cuticle (setae and microsculpturing) bear some structural resemblance to the ultrablack surfaces in other animals [10,13]. Moreover, there are lamellae underneath the epicuticle, stacked on top of each other and interconnected by columns. This kind of structure can also be found in another wasp, the oriental hornet *Vespa orientalis* (Hymenoptera, Vespidae) [20–22], however, it does not result in ultrablack colors.

The structures of *T. bifurca* may act as a light-trapping mechanism and can enhance absorption by 5% compared to a flat surface. The dense layer of black epicuticular setae further enhances ultrablack coloration. Ishay and collaborators [22] suggested that longitudinal chitinous plates would be interconnected by dark transverse and longitudinal absorptive bands. These are often rods of chitin embedded in a protein matrix [23]. The authors also suggested that there is a proteinaceous filling material, which tends to leach out during preparation of the specimens for SEM, leaving behind empty lacunae. Modelled reflectance of *V. orientalis* suggests that this layered structure contributes to the overall antireflective properties of the cuticle by increasing the effective surface area available for light absorption [20]. Although this system in *V. orientalis* is coupled with xanthopterins to absorb light, the similar structure in *T. bifurca* is most probably coupled with melanin as in other velvet ants [24].

Similar cases of white–black structural colors can be observed in other insect groups. For instance, the tiger mosquito *Aedes albopictus* (Diptera: Culicidae) exhibits black and white scales with micro- and nanostructures that turn transparent scales into superwhite and melanized scales into superblack [25]. Melanin pigments can produce a black color with visible reflections, such as in shiny fur and feathers. The degree of such visible reflections depends on the surface structure. Ultrablack color lacks this gloss and instead displays a matte black appearance due to the presence of (often hierarchical) surface microstructures. For example, in the beetle *Alaus oculatus* (Coleoptera: Elateridae), there are two large dorsal circular spots known as eyespots. Within these spots, fixed setae are present in a concave structure, and the underlying cuticle features a microstructured topography [26]. These concave structures scatter light and enhance absorption by melanin, serving as an antireflective feature and creating ultrablack colors [26]. In another beetle species, *Euprotaetia inexpectata* (Coleoptera: Scarabaeidae), a complex ultrastructure consisting of randomly oriented anisotropic micropillars accounts for its significant absorptivity [27].

Peacock spiders feature microstructures in specific body regions that exhibit ultrablack coloration with high absorbance. Interestingly, these microstructures are absent in other dark body areas with lower UV–vis absorbance [10]. These microstructures scatter light across the absorptive cuticle, further aided by brush-like scales that serve as an anti-reflective coating [10]. Similarly, birds-of-paradise display ultrablack coloration attributed to microstructures found in the barbules of their feathers, yielding a similar effect [13]. West African Gaboon vipers exhibit dorsal patches of black scales with microscale leaf-like elevations and nanoscale ridges that result in ultrablack colors [14–15]. These examples suggest that microstructure-assisted ultrablack colors are naturally selected and may have evolved convergently across animal groups, including velvet ants.

In addition to their role in color production, submicrometer-sized structures, when randomly distributed in size and position on (or in) a transparent material, can enhance light scattering. This scattering can intensify white coloration; however, when the underlying material is black, as in *T. bifurca*, these structures can contribute to the enhancement of ultrablack coloration by amplifying light absorption. Future studies should investigate this phenomenon in greater detail, exploring how variations in the distribution and organization of such microstructures influence the absorption efficiency and potential adaptive significance of ultrablack coloration in different ecological contexts.

The role of ultrablack colors in nature is still a topic of debate, with limited evidence regarding the selective pressures driving their evolution. Recent studies propose that ultrablack coloration may enhance visual signals from bright colors in peacock spiders and birds-of-paradise, traits that are sexually selected [10,13]. However, in velvet ants, sexual behavior in most species remains largely unexplored, and the sparse evidence suggests little correlation between female coloration and male preference [28]. As a result, an alternative hypothesis regarding the evolution and function of ultrablack colors in velvet ants is the amplification of antipredatory visual signals.

Female velvet ants are renowned for their effective defenses against predators. Given their characteristics and life habits, such as diurnal activity, striking colors, and inability to fly, it is expected that they would be frequent targets for predators. However, there is limited observational and experimental evidence documenting interactions between velvet ants and insectivorous predators [29–30]. Observations indicate that while bufonid toads may initially prey on female velvet ants, they tend to avoid them in subsequent encounters [29–30]. Conversely, birds and lizards, which are known predators of defended insects such as bees and wasps, exhibit caution and generally avoid interacting with female velvet ants [30–31]. Therefore, this behavior suggests that the colors of velvet ants, closely linked to their defense mechanisms, such as cuticle hardness and their painful sting, may function as an honest warning signal to potential predators.

Thermal imaging suggests that the body temperature of *T. bifurca* in a range of ambient temperatures generally remains below ambient temperature. There is evidence indicating that structures like the long white setae in Thistledown velvet ants (*Dasymutilla gloriosa*) in the Desert Mimicry Ring may play a role in thermal regulation [32]. Additionally, previous studies have suggested a potential association between dark colors in velvet ants and photoprotection [16]. In this case, the dark cuticle would function as a radiation filter to prevent ultraviolet radiation from reaching the cells underneath. This protection may derive from melanin [16], and the tile-shaped cuticular lamellae that might protect from the damage of exposure to UV light [33]. Our findings do not provide compelling evidence regarding the thermal implications of ultrablack colors in *T. bifurca*, even though the wasps were always 2 °C below ambient temperatures. Surprisingly, the white setae did not exhibit distinct thermal behavior compared to the dark colors. Furthermore, the analysis of setae ultrastructures revealed no significant differences among them. Therefore, future investigations should address not only the thermal implications of ultrablack colors compared to other velvet ants with non-ultrablack dark colors, but also the thermal properties of white setae.

Another potential function of the sculptured cuticle is resistance to high forces. Velvet ants are known as “indestructible insects” not only because most predators fear their panful stings and venom, but also because of their hard exoskeleton [29–30]. The sculptured cuticle may have a similar structure and force-resisting mechanisms as other wasps [34]. In some wasp species, the sculpturing of the cuticle and the lamellae terraces may form an accordion-like structure that increase resistance to fractures and high pressures [34].

Ultrablack coloration has garnered significant interest in recent years because of its potential applications across various fields, including optics, camouflage, and solar energy capture. Characterized by their remarkable ability to reflect an exceptionally low amount of light across a broad spectrum of wavelengths in the UV–vis range, these surfaces have drawn inspiration from natural occurrences observed in certain organisms. Scientists are actively engaged in deciphering the underlying mechanisms behind the development of these ultrablack surfaces, with the goal of replicating such structures in synthetic materials possessing analogous properties [3,5–6,15].

In conclusion, the study of ultrablack coloration in animals, such as *T. bifurca*, reveals the intricate interplay between structural microfeatures and pigment absorption that results in these remarkably absorptive surface. Especially, this kind of wasp-inspired technology may have its application in increasing efficiency of solar panels [20]. Further research is needed to uncover the mechanisms and functional roles of ultrablack coloration in velvet ants, as well as to determine which other Hymenopteran species exhibit this fascinating color trait.

## Supporting Information

File 1 Spectrum obtained from females of *Traumatomutilla bifurca*.

## Data Availability

All data that supports the findings of this study is available in the published article and/or the supporting information of this article.
